# Health literacy and high blood pressure among Myanmar migrant workers in Northeastern Thailand

**DOI:** 10.1371/journal.pone.0302057

**Published:** 2024-04-25

**Authors:** Kittipong Sornlorm, Wor Mi Thi

**Affiliations:** Faculty of Public Health, Khon Kaen University, Nai Mueang, Mueang Khon Kaen, Khon Kaen, Thailand; Ministry of Health, MYANMAR

## Abstract

Hypertension, related to serious consequences unless diagnosed and treated, is a global concern, also affecting migrant workers. Due to the high prevalence of hypertension among migrant workers in Thailand, the influences of health literacy and other factors on blood pressure were needed to explore among Myanmar migrant workers in Northeastern Thailand. Hence, this study aimed to identify the prevalence of high blood pressure (HBP), its association with health literacy and other factors among Myanmar migrant workers in Northeastern Thailand. A cross-sectional analytical study was conducted in Northeastern Thailand. A multistage sampling procedure was applied. Data was gathered through interviews utilizing validated and reliable structured questionnaire. Descriptive statistics and inferential statistics with multiple logistic regression were used. Among 406 participants, about 60% were male and about 70% of them were married. The prevalence of overall HBP was 63.3% (95% CI: 58.49–67.86), 52% in females and 71.86% in males. Participants with limited health literacy were 79% more likely to have HBP than those with excellent and adequate health literacy (AOR = 1.79, 95% CI: 1.13–2.84). Other factors substantially linked with having HBP were being male gender (AOR = 4.68, 95% CI: 2.81–7.78), being overweight (AOR = 2.23, 95% CI: 1.18–4.23), being obese (AOR = 5.69, 95% CI: 2.96–10.96), not having health insurance (AOR = 2.01, 95% CI: 1.11–3.66), staying in Thailand for more than 48 months (AOR = 2.4, 95% CI: 1.48–3.9), and having family history of hypertension (AOR = 2.07, 95% CI: 1.28–3.35). In conclusion, more than half of Myanmar migrant workers had HBP. Factors associated with HBP were limited health literacy, male gender, overnutrition, lack of health insurance, longer duration of stay in Thailand and having family history of hypertension. Therefore, there is a need for a multifaceted strategy to prevent hypertension and its consequences by promoting health literacy as well as by enhancing good behavioural practice among this community.

## 1. Introduction

Hypertension spurred premature death contributed to 23% of morbidity and 68% of global deaths but being still underdiagnosed and treated [1]. It linked to cardiovascular, cerebrovascular [2,3], renal [4], cognitive, and dementia risks [5]. Its prevalence doubled globally from 1990 to 2019 [6]. Pre-hypertension is a condition where the blood pressure is higher than normal [7], and which can later be progressed into hypertension within 2 to 4 years [8,9]. In age ≥18 years, Myanmar’s hypertension rate was 23% and Thailand’s 25% [10]. In Northeastern Thailand, hypertension prevalence among adult (> 15 years old) was 21% [11].

Migrant workers faced extra risks due to migration and lifestyle changes [11,12]. Thailand is a country which hosted 1.5 million Myanmar migrant workers in 2020 [13]. According to Department of Employment (DOE), in October 2022, 32% of total 60,319 migrant workers in Northeastern Thailand were comprised of Myanmar migrant workers [14]. Among Myanmar migrant workers (18–60 years) in Thailand, a study showed that 27.8% were hypertensive [15] and another study showed that 68.3% of them had pre-hypertension [16].

It was proven that inadequate health literacy is highly associated with prevalence of hypertension [17–19]. Health literacy was defined as the personal abilities to acquire, process, and comprehend health information and services required to make wise decisions about one’s health [20]. In addition to health literacy, other associated factors for hypertension were age, sex, alcohol drinking and being overweight or obese [15,21]. Moreover, socioeconomic status, education status, lifestyle, diet style, and certain ethnicity were also associated with hypertension [22].

Although some studies explored the prevalence of pre-hypertension and hypertension among Myanmar migrant workers in Thailand, the socioeconomic status, behavioral practices, and the factors determining health might vary depending on living environment and from region to region. Moreover, there is a need to discover the health literacy status and its association to high blood pressure (HBP) which is a potential condition to suffer from hypertension related consequences unless discover and apply necessary actions among Myanmar migrant workers in Thailand. Therefore, this study aimed to explore HBP prevalence and its association to health literacy and other factors among Myanmar migrant workers in Northeastern Thailand.

## 2. Materials and methods

### 2.1 Study design and sampling procedure

This study was cross-sectional analytical study conducted in Khon Kaen Province by interviewing 406 Myanmar migrant workers using structured questionnaire between 25^th^ June 2023 to 28^th^ August 2023. Required sample size was estimated by logistic regression formula of Hseih, Bloch, and Larsen in 1998 [23], where P0 was 0.19, P1 was 0.41 and B was 0.41, estimated based on previous study [15]. Preliminary questions were asked with inclusion and exclusion criteria before taking consent to be accounted as potential participants. Inclusion criteria were registered migrant workers, had been working in Northeastern Thailand for at least three months duration, able to communicate with Myanmar language, gave informed consent, willing to participate and age from 18–59 years old. Those with serious bedridden illness, those who were on antihypertensive medications for more than six months and those with pregnancy were excluded.

The samples were chosen by multistage sampling procedure. Firstly, we chose top three provinces with highest number of Myanmar migrant workers among the 20 provinces from Northeastern Thailand. They were Nakhon Rachsima province, Khon Kaen province and Chiya Phum province. Among them, Khon Kaen province was selected by simple random sampling method. As of October 2022, there were 3473 Myanmar migrant workers working in Khon Kaen Province [14]. About 68% of them were working in 5 fishing net factories and one construction site in Khon Kaen Provinces who were living around their respective workplace as communities. So, we considered those communities due the highest distribution of Myanmar migrant workers that was significant to represent the targeted population. We contacted and invited migrant workers through the respective community leaders to participate in the study. Migrant workers from communities living around one factory (1200 population) and the community living in the construction camp site (300 population) responded to participate in the study. The required sample size was selected by population proportion to size technique (325 participants from community living around the factory and 81 participants from community living in the construction camp site). Firstly, the house numbers of the Myanmar migrant workers were listed. Index case was chosen from the first three house number by simple random method. Then the samples were collected by systematic random sampling procedure. If there were two potential participants willing to participate in the same household, we selected only one participant by lottery method among them to avoid bias. When there was no response person in the selected house, we left that number and continued data collecting according to procedure until finished. Then, we added the required sample starting from the end house number of previous procedure. The non-response rate in our study was two percent. We collected the samples considering non-response rate until the required sample size was reached.

### 2.2 Study outcome

Blood pressure of the participant was measured for 2 times with 5 minutes apart following the instructions of “Seventh Report of Joint National Committee (JNC7)” by validated automated oscillometer upper-arm cuff device with appropriate cuff size which covered at least 80% of arm and calculated for average value [7]. At first, blood pressure was classified into four groups based on Systolic Blood Pressure (SBP) and Diastolic Blood Pressure (DBP); (1) Normal blood pressure (SBP <120 mmHg and DBP <80 mmHg); (2) Pre-hypertension (SBP 120–139 mmHg and/or DBP 80–89 mmHg); (3) Stage one hypertension (SBP 140–159 mmHg and/or DBP 90–99 mmHg); (4) Stage two hypertension (SBP ≥160 mmHg and/or ≥100mmHg). Then, HBP was considered when the average SBP of at or above 120 mmHg and/or DBP of at or above 80 mmHg.

### 2.3 Data collection tools and techniques

A structured questionnaire consisted of eight parts was used: (1) Socio-demographic characteristics such as age, sex, marital status, educational status, monthly individual income (Baht/Month), monthly individual expenditure (Baht/Month), health insurance and duration of stay in Thailand; (2) Behavioural factors such as tobacco use (either smoking or betel chewing or tobacco chewing), alcohol use, physical activity, sleep duration (Hour/Day), working hour (Hour/Day), consumption of salt and salty diet (Number of days/Week), consumption of oil (Type of most often used oil), consumption of fruit and vegetables (Number of day of having at least 5 servings/Week); (3) Physical health status such as perceived health status, history of chronic illness, family history of hypertension, family history of NCDs; (4) Mental health status such as depression disorders and stress; (5) Health literacy; (6) Knowledge on hypertension; (7) Attitude towards hypertension; (8) Physical measurements such as blood pressure, weight and height for Body Mass Index (BMI) and waist circumference.

Adapted version of HLS Asia questionnaire updated with hypertension information was used [24]. The "inadequate" and "problematic" levels were consolidated into a single level dubbed "limited health literacy" in order to identify vulnerable groups [25]. Depression disorder was measured by nine items Patient Health Questionnaire (PHQ9) [26], and stress was measured by Perceived Stress Scale (PSS) [27]. Knowledge of hypertension and its complication were asked in line with Bloom’s theory [28]. Attitude towards hypertension were asked covering affective domains according to Bloom’s theory [29]. BMI classification for the Asia-Pacific region was used [30]. The World Health Organization (WHO) interim cut off points for Asia Pacific waist circumference values were applied [30].

Content validity was checked by three expertise and the questionnaire was revised accordingly. Item Objective Congruence (IOC) of 0.80–1.00 for each item was obtained. The questionnaire was translated forward and backward between English and Myanmar and was certified by expertise in the related field. Pre-test was done to 30 Myanmar migrant workers who were not the participants of this study. Then, the questionnaire was adjusted accordingly. Cronbach’s alpha coefficient was 0.85 for health literacy, 0.88 for knowledge, 0.77 for attitude. Three data collectors from the public health field were trained in person for one day and standardized.

### 2.4 Statistical analysis

After verification, the data was recorded in Microsoft excel and then imported into STATA program, version 15, Texas USA, serial number of 301506215585. Descriptive statistics and inferential statistics were used. Categorical data were described by frequency count and percentage while continuous data were described using mean, standard deviation (SD), median, minimum, and maximum. Bivariate analysis using simple logistic regression was done initially with each factor and HBP. Factors that had p-value less than 0.25 after bivariate analysis which were included in the initial model of multiple logistic regressions involved age, gender, educational attainment, health insurance, duration of stay in Thailand, most used oil in last month, consumption of at least five servings of fruits and vegetables in a week, tobacco use, alcohol use and working duration, BMI, waist circumference, perceived health status, having chronic disease, family history of hypertension and family history of chronic disease and health literacy. Multicollinearity was tested and VIF value was 1.38. Multivariable analysis by multiple logistic regression with backward elimination method was used. The association was described with adjusted odds ratio (AOR) and its 95% confidence interval (95% CI). p-value of less than 0.05 was considered statistically significant. Model fitness was tested by goodness-of-fit test and p-value was 0.57. Therefore, under this model, the observed data adequately fits the expected distribution. Moreover, the area under receiver operating characteristics curve (ROC) was resulted as 0.746. Hence, the model could predict the outcome correctly and could be inferenced to the population.

### 2.5 Ethical consideration

This study was approved by “Khon Kaen University Ethics Committee for Human Research Ethic” based on the Belmont Report and GCP in Social and Behavioral Research with the reference number HE662091. All the participants were ensured to be volunteers and informed written consent was obtained from every participant before participation. The confidentiality of the participants was taken as the priority, and the identity was not revealed by name or in other ways. Only identification numbers were used. Soft copy of the data was stored by protecting password. Hard copies of the data were kept in a locked drawer.

## 3. Results

### 3.1 Baseline characteristics and prevalence of high blood pressure (HBP) among Myanmar migrant workers in Northeastern Thailand

#### 3.1.1 Sociodemographic characteristics

Sociodemographic characteristics were described in Table 1. The highest proportion (44.83%) of the 406 Myanmar migrant workers were under the age of 30, with an average age of 32.28 years. More than half of the participants were male (56.9%), most of them were married (67.98%). About one third of participants (31.77%) attained high school level of education and only 3.46% of them reached the bachelor and above level. Their average monthly income was around 10,000 Thai Baht, average monthly expense was 3525.86 Thai Baht and 80.3% of them had health insurance. The typical duration of their stay in Thailand was 48 months.

**Table 1:** Sociodemographic characteristics of Myanmar migrant workers in Northeastern Thailand (n = 406).

Characteristics	Number (n)	Percentage (%)
**Age (completed years)**		
<30	182	44.83
30–39	131	32.27
40–49	73	17.97
≥50	20	4.93
Mean(±SD)	32.28(±9.29)	
Med (Min:Max)	31(18:56)	
**Gender**		
Male	231	56.90
Female	175	43.10
**Marital status**		
Single	130	32.02
Married	276	67.98
**Educational attainment**		
Illiterate	7	1.72
Primary school	99	24.38
Middle school	157	38.67
High school	129	31.77
Bachelor and above	14	3.46
**Monthly income (Thai Baht)**		
≤10000	271	66.75
>10000	135	33.25
Mean(±SD)	10273.40(±1747.05)	
Med (Min:Max)	10000(4000:25000)	
**Monthly expense (Thai Baht)**		
≤3000	226	55.67
>3000	180	44.33
Mean(±SD)	3525.86(±1518.97)	
Med (Min:Max)	3000(1000:10000)	
**Having health insurance**		
Yes	326	80.30
No	80	19.70
**Duration of stay in Thailand (months)**		
≤48	216	53.20
>48	190	46.80
Mean(±SD)	59.93(±61.45)	
Med (Min:Max)	48(3:360)	

*n = number of samples.

#### 3.1.2 Behavioural factors and physical health status of participants

Table 2 presents behavioral factors and physical health status of Myanmar migrant workers. The most frequently used oil in the previous month was determined using locally available and widely consumed oils. Soybean oil was consumed by more than one-third of Myanmar migrant workers, while ground nut oil was consumed by another one-third (38.67% and 33.74% respectively). However, consumption of instant food/fast food, as well as the use of added salt, were low, with less than 5 days of use per week (6.65% and 11.08%, respectively). Similarly, one-third of them (31.03%) ate a salty diet on most of the days (at least five days) in a week. While, only 22.17% met the requirement of eating at least 5 servings of fruits and vegetables on most days of the week (5 days or more). More than half of them had used tobacco at some point in their lives, with 44.58% being current users and 11.58% being former users. Similarly, about half of them drink alcohol at some point in their lifetime with 31.77% of current drinkers and 20.94% of former drinkers. Physical activity was measured using the WHO-STEPS questionnaire, which asked about physical activities at work, transportation, and leisure time and classified them as adequate or inadequate based on WHO recommendations. More than 80% of the individuals (83.5%) reached the WHO requirement for appropriate physical activity, while less than 20% (16.5%) did not meet the recommendation. The average length of continuous sleeping at night in hours has been asked. About half of them (52.22%) slept for 8 hours or more per night, with an average of 6.46 (±1.59) hours, whereas the lowest sleeping time was 1 hour, and the longest sleeping duration was 12 hours. 62.56% of participants had working hours of 10 or more, with an average working hour of 9.51(±1.56).

**Table 2:** Behavioral factors and physical health status of Myanmar migrant workers in Northeastern Thailand (n = 406).

Characteristics	Number (n)	Percentage (%)
**Behavioral factors**		
**Most used oil in last month**		
Soybean oil	157	38.67
Ground nut oil	137	33.74
Mixed groundnut oil and palm oil	50	12.33
Palm oil	23	5.66
Sunflower oil	16	3.94
Other	23	5.66
**Consumption of instant and fast food per week (days)**		
< 5	379	93.35
≥ 5	27	6.65
**Consumption of added salt per week (days)**		
< 5	361	88.92
≥ 5	45	11.08
**Consumption of salty diet per week (days)**		
< 5	280	68.97
≥ 5	126	31.03
**Consumption of at least 5 servings of fruits and vegetables in a week (days)**		
< 5	316	77.83
≥ 5	90	22.17
**Tobacco use**		
Non- user	178	43.84
Former user	47	11.58
Current user	181	44.58
**Alcohol use**		
Non-drinker	192	47.29
Former drinker	85	20.94
Current drinker	129	31.77
**Physical activity**		
Met WHO recommendation	339	83.50
Unmet WHO recommendation	67	16.50
**Sleep duration at night (Hours)**		
<8	194	47.78
≥8	212	52.22
Mean(±SD)	6.46(±1.59)	
Med (Min:Max)	7(1: 12)	
**Working duration (Hours)**		
≤8	133	32.76
>8	273	67.24
Mean(±SD)	9.51(±1.56)	
Med (Min:Max)	10(4:14)	
**Physical health status**		
**Body Mass Index (BMI) (kg/m2)**		
Underweight (<18.5)	47	11.58
Normal (18.5–22.9)	197	48.52
Overweight (23–24.9)	67	16.50
Obesity (≥25)	95	23.40
**Waist circumference (cm)**		
Below interim cut off point (<90 in men & <80 in women)	311	76.60
Interim cut off point and above (≥90 in men & ≥80 in women)	95	23.40
**Perceived health status**		
Healthy	87	21.43
Unhealthy	319	78.57
**Having chronic disease**		
No	343	84.48
Yes	63	15.52
**Family history of hypertension**		
No	249	61.33
Yes	157	38.67
**Family history of chronic disease**		
No	345	84.98
Yes	61	15.02

Many people in the study population were overnutrition with overweight (16.50%) and obese (23.40%). However, in terms of waist circumference, the majority (76.6%) fell below WHO’s interim cut-off point of less than 90cm in males and less than 80cm in females. Most of them (78.57%) perceived as being unhealthy at the time of data collection. Among participants, 15.52% of them reported as having a chronic condition. When we queried the participants about their family history, 38.67% had a family history of hypertension and 15.02% had a family history of chronic illnesses.

#### 3.1.3 Mental health status, health literacy, knowledge, and attitude of participants

Mental health status, health literacy, knowledge, and attitude of the Myanmar migrant workers were presented in Table 3. Among the participants, the majority (71.43%) had moderate stress while 4.68% had high levels of perceived stress. Most of the participants (47.54%) did not have depressive disorder followed by mild depressive disorder (29.06%) at the time of data collection. About one third of the participants had problematic health literacy (34.48%) whereas 21.67% were inadequate in health literacy. About 45% of them had low level knowledge and more than half of the participants (56.16%) had good attitude towards hypertension.

**Table 3:** Mental health status, health literacy, knowledge, and attitudes of Myanmar migrant workers in Northeastern Thailand(n = 406).

Characteristics	Number (n)	Percentage (%)
**Mental health status**		
**Perceived stress (PSS)**		
Low (0–13)	97	23.89
Moderate (14–26)	290	71.43
High (27–40)	19	4.68
Mean scores (±SD)	17.26(±5.95)	
Median scores (Min:Max)	18(0:36)	
**Depressive disorder (PHQ9)**		
None (0–4)	193	47.54
Mild (5–9)	118	29.06
Moderate (10–14)	47	11.58
Moderately severe (15–19)	38	9.36
Severe (20–27)	10	2.46
Mean scores (±SD)	6.42 (±5.56)	
Median scores (Min:Max)	5(0:27)	
**Health literacy**		
Excellent (>84%)	38	9.36
Adequate (>66% - 84%)	140	34.49
Problematic (>50% - 66%)	140	34.48
Inadequate (≤50%)	88	21.67
Mean health literacy scores (±SD)	60.62(±15.35)	
Median scores (Min:Max)	61(24:96)	
**Knowledge**		
Low (<60%)	182	44.83
Average (60% - 79%)	134	33.00
High (≥ 80%)	90	22.17
Mean knowledge scores (±SD)	6.58 (±2.57)	
Median knowledge scores (Min:Max)	7 (0:11)	
**Attitude**		
Poor (<60%)	19	4.68
Moderate (60% - 79%)	159	39.16
Good (≥ 80%)	228	56.16
Mean attitude scores (±SD)	36.17 (±5.29)	
Median attitude scores (Min:Max)	36 (24:45)	

*n = number of samples.

#### 3.1.4 Prevalence of HBP among Myanmar migrant workers in Northeastern Thailand

Blood pressure was measured two times and average blood pressure was taken according to JNC 7 guideline. The prevalence of high blood pressure was 63.3% (95% CI: 58.49–67.86) among Myanmar migrant workers with the blood pressure level of pre-hypertension (36.45%,95% CI: 31.89–41.26), stage 1 hypertension (20.45%, 95% CI: 16.78–24.65) and stage 2 hypertension (6.4%, 95% CI: 4.39–9.24).

### 3.2 Level of health literacy and prevalence of HBP

The prevalence of HBP by level of health literacy in different health domains and health literacy dimensions were shown in Table 4. The highest HBP prevalence was seen in problematic group by dimension of apply (76.47%) where the lowest HBP prevalence was seen in excellent groups by dimension of access (54.84%) and health care domain (54.84%).

**Table 4:** Level of health literacy and prevalence of HBP by different health literacy dimensions and different health domains among Myanmar migrant workers in Northeastern Thailand (n = 406).

Level of health literacy	Number of samples	High blood pressure		95% CI
		n	%	
**Health literacy by dimension of access**				
Excellent (>84%)	62	34	54.84	42.37–66.73
Adequate (>66% - 84%)	120	75	62.50	53.50–70.71
Problematic (>50% - 66%)	65	40	61.54	49.22–72.54
Inadequate (≤50%)	159	108	67.92	60.25–74.73
**Health literacy by dimension of understand**				
Excellent (>84%)	49	32	65.31	51.06–77.25
Adequate (>66% - 84%)	140	83	59.29	50.94–67.12
Problematic (>50% - 66%)	108	73	67.59	58.20–75.75
Inadequate (≤50%)	109	69	63.30	53.86–71.83
**Health literacy by dimension of appraise**				
Excellent (>84%)	67	41	61.19	49.07–72.07
Adequate (>66% - 84%)	130	83	63.85	55.23–71.66
Problematic (>50% - 66%)	89	61	68.54	58.17–77.34
Inadequate (≤50%)	120	72	60.00	50.98–68.39
**Health literacy by dimension of apply**				
Excellent (>84%)	87	53	60.92	50.29–70.59
Adequate (>66% - 84%)	151	87	57.62	49.58–65.27
Problematic (>50% - 66%)	85	65	76.47	66.28–84.31
Inadequate (≤50%)	83	52	62.65	51.78–72.38
**Health literacy by health care domain**				
Excellent (>84%)	62	34	54.84	42.37–66.73
Adequate (>66% - 84%)	93	55	59.14	48.88–68.66
Problematic (>50% - 66%)	147	100	68.03	60.05–75.08
Inadequate (≤50%)	104	68	65.38	55.74–73.91
**Health literacy by disease prevention domain**				
Excellent (>84%)	67	43	64.18	52.06–74.73
Adequate (>66% - 84%)	101	58	57.43	47.60–66.70
Problematic (>50% - 66%)	137	89	64.96	56.60–72.50
Inadequate (≤50%)	101	67	66.34	56.57–74.88
**Health literacy by health promotion domain**				
Excellent (>84%)	71	43	60.56	48.79–71.22
Adequate (>66% - 84%)	116	70	60.34	51.17–68.85
Problematic (>50% - 66%)	123	82	66.67	57.86–74.44
Inadequate (≤50%)	96	62	64.58	54.52–73.50

*n = number of samples, 95% CI = 95% Confidence Interval.

### 3.3 Factors associated with HBP among Myanmar migrant workers

In bivariate analysis, 10 factors were statistically associated with higher odds of HBP (P-value<0.05) which were age, gender, more than 48 months of staying in Thailand, consumption of at least five servings of fruits and vegetables less than 5 days in a week, tobacco use, alcohol drinking, BMI (overweight and obesity), bigger waist circumference, having chronic disease, and having family history of hypertension. Seven factors had p-value < 0.25 which were educational attainment, health insurance, most used oil in last month, working duration, perceived health status, family history of chronic disease and health literacy. Therefore, a total of 17 factors were included into the initial model of multivariable analysis after checking multicollinearity between them.

Table 5 shows factors associated with HBP among Myanmar migrant workers in Northeastern Thailand. After controlling other covariates in multiple logistic regression, health literacy was associated with HBP. Myanmar migrant workers who had limited level of health literacy were 79% more likely to be HBP than those with excellent health literacy (AOR = 1.79, 95% CI: 1.13–2.84). In terms of gender, male had 4.68 times higher odds of having HBP than female (AOR = 4.68, 95% CI: 2.81–7.78). Those who did not have health insurance were 2 folds more likely to have HBP than those who had health insurance (AOR = 2.01, 95% CI: 1.11–3.66). Longer duration of stayed in Thailand was also associated with having HBP. Among the participants, those who stayed in Thailand for more than 48 months have the significantly higher risks of being HBP than those who stayed only up to 48 months (AOR = 2.40, 95% CI: 1.48–3.90). Our study also found out the association of nutritional status to HBP. Overweight participants had more than 2 folds (AOR = 2.23, 95% CI: 1.18–4.23), obese participants had 5.69 times (AOR = 5.69, 95% CI: 2.96–10.96) higher risks of being HBP than normal and underweight participants. In addition, we found a substantial link with family history to HBP. Among respondents, those who had family history of hypertension were 2 times more likely to have HBP than those who did not have family history (AOR = 2.07, 95% CI: 1.28–3.35).

**Table 5:** Multivariable analysis of health literacy and factor associated with HBP using multiple logistic regression among Myanmar migrant workers in Northeastern Thailand (n = 406).

Factors	Number of samples	High blood pressure		COR	AOR	95% CI	p-value
		n	%				
**Health literacy**							0.014
Excellent & adequate (>66%)	178	104	58.43	1	1		
Limited (≤66%)	228	153	67.11	1.45	1.79	1.13–2.84	
**Sociodemographic characteristic**							
**Gender**							<0.001
Female	175	91	52.00	1	1		
Male	231	166	71.86	2.36	4.68	2.81–7.78	
**Having health insurance**							0.022
Yes	326	199	61.04	1	1		
No	80	58	72.50	1.68	2.01	1.11–3.66	
**Duration of stay in Thailand (months)**							<0.001
≤48	216	123	56.94	1	1		
>48	190	134	70.53	1.81	2.40	1.48–3.90	
**Physical health status**							
**Body Mass Index (BMI) (kg/m2)**							<0.001
Normal & underweight (<23kg/m2)	244	134	54.92	1	1		
Overweight (23–24.9kg/m2)	67	46	68.66	1.80	2.23	1.18–4.23	
Obesity (> 25 kg/m^2)^	95	77	81.05	3.51	5.69	2.96–10.96	
**Family history of hypertension**							0.003
No	249	146	58.63	1	1		
Yes	157	111	70.70	1.70	2.07	1.28–3.35	

*n = number, COR = Crude Odds Ratio, AOR = Adjusted Odds Ratio, 95% CI = 95% Confidence Interval.

## 4. Discussion

### 4.1 Prevalence of HBP among Myanmar migrant workers in Northeastern Thailand

In this study, the prevalence of HBP was 63.3% with 52% in females and 71.86% in males. The prevalence was notable and consistent with a previous study in Surat Thani Province, which was 68.3% with the same blood pressure cut off point as our study among Myanmar migrant workers (18–59 years) in Thailand [16]. This demonstrated that if Myanmar migrant workers were ignored, they were at greater risk of developing hypertension and accompanying consequences. Furthermore, the prevalence was substantially greater than a Chinese study conducted among female migrant workers (18–45 years) with a blood pressure cut off point of SBP 130mmHg and/or DBP 80 mmHg, where the prevalence of HBP was 27.2% [21]. There were some differences in blood pressure cut off point between this study and our study.

Furthermore, according to JNC 7, the proportion of pre-hypertension in this study was 36.45%, which was consistent with 35.15% in Chinese adults (18–70 years) [31]. Furthermore, we discovered that the proportion of hypertension was 26.85%, with 20.45% being stage one hypertension and 6.4% being stage two hypertension. This was marginally lower than the previous study’s result of 27.8% of hypertensin among Myanmar migrant workers (18–60 years) in Chiang Mai, Northern Thailand [15]. As an individual behavior can be affected by surroundings, the higher consumption of sodium intake in Northern Thailand than Northeastern Thailand [32] can have effects on the blood pressure of individuals residing in that area. But, the proportion of hypertension among Myanmar migrant workers was higher than the national prevalence of host country of Thailand; 25% in adults 18 years and older [10], and much higher than the national prevalence of home country of Myanmar; 23% in adults 18 years and older [10], as well as higher than middle age and elderly adult of Myanmar (≥40 years); 21.95% [24]. This could be owing to the added hazards of adding new behavioral risk factors to the current risk factors carried by migrants from their home country, as well as any restraints that may prevent them from seeking health care [12].

### 4.2 Association of health literacy and other factors with HBP

In our study, the proportion of overall limited hypertensive health literacy was 56.15%, which was slightly lower than the nation of origin (Myanmar), which was 60% among people aged 40 and above [24]. This could be because of the media illiteracy, language barrier, and financial barrier in accessing health information and health services. Moreover, poor knowledge and perception in this population might lead them to face difficulties in applying the health information. However, health literacy among Myanmar migrant workers in Thailand was higher than the migrant workers in Saudi Arabia where they had only 13.38% of adequate and excellent health literacy [33]. After controlling other covariates, limited health literacy increased the risk of having HBP by 79%. Therefore, this study found out that the higher the health literacy, the lower the odds to be high in blood pressure. This protective association of health literacy for hypertension from our study was supported by; a study in Japan [19], a Brazil study [34], an Iran Study [35], and a Croatia study [18].

Factors such as aging [1,15,22,36], less consumption of fruits and vegetables at least five servings of fruits [1], tobacco use [37], alcohol use [15,36,38], bigger waist circumference [37,39,40], having chronic disease [41] were known risk factors for hypertension. These factors were also associated with higher odds of HBP only in bivariate analysis but not associated in multivariable analysis of this study. Although there was no association between HBP and tobacco or alcohol consumption in the final model, the connection observed in the bivariate analysis, along with the notably high proportion of tobacco use and alcohol consumption among Myanmar migrant workers, implies that these factors are crucial when devising strategies to mitigate hypertension and its consequences.

Multivariable analysis of our study found that being male were significantly higher to have HBP than being female. This finding was consistent with several studies conducted; in Ethiopia [42], among Myanmar migrant workers in Thailand [15], a systematic review [42], and a guidelines and policies review of China [43]. This could be because male participants from our study practiced behavioral risk factors such as alcohol drinking and smoking more than female participants. Moreover, our study found that not having health insurance increased the risk of having high blood pressure. The financial barrier resulting from out of pocket might lead them to hesitate to seek health care. Therefore, to prevent, identify and treat NCDs it is crucial to ensure that everyone is accessible to the universal health care by any means [12]. Our study also pointed out being overweight and obese were highly associated with high blood pressure. Overweight and obesity are the condition called overnutrition which occurs when supplying nutrients exceeds the requirement so that the excess nutrients are stored as fat in our body [44]. The status of overnutrition (overweight/obesity) increases the risk of metabolic syndrome including type 2 diabetes mellitus, hypertension, hyperlipidemia etc. by attributing various functional changes in the body [44]. The result from our study was in accordance with findings from other studies where being overnutrition (overweight and obesity) increased the odds of having hypertension [1,15,16,21,22,40]. Being overweight and obesity were associated not only with blood pressure but also with other NCDs which have interaction with blood pressure [10]. Therefore, weight control was an important thing to consider in this group to prevent hypertension and its related complications as well as developing from other NCDs. In addition, longer duration of stay in Thailand had a greater risk of having HBP. The difficulties of migrant workers in adjusting to a new culture might lead them to adopt hazardous lifestyle practices on top of their pre-existing risks [12]. Our finding was supported by previous study where the Myanmar migrant workers with working experience of at least 48 months were more likely to have pre-hypertension [16]. Likewise, our study also found that Myanmar migrant workers with family history of hypertension were more likely to be high in blood pressure. This could be not only due to the fact of genetic linkage [45], but also used to engaging and inheriting the unhealthy environment as well as unhealthy life style choices such as eating habits, smoking, alcohol drinking and not doing physical exercises.

To address the high prevalence of HBP in the Myanmar migrant population and prevent its direct and indirect repercussions, effective intervention techniques concentrating on prevention, precise diagnosis, and timely treatment are necessary. Health promotion programs on health literacy promotion, particularly for hypertension and other noncommunicable diseases (NCDs), using multilingual health education materials to promote community understanding and acceptance should be enhanced. Furthermore, identifying and closing gaps in access to universal health coverage is critical in applying and accessing health information and health services. Additionally, adopting food safety policies and creating recreational places can aid in the fight against overnutrition. Additional research is required to investigate the causality of risk factors in relation to hypertension such as cohort studies and to identity the association between level of health literacy and medication adherence as well as controlled of blood pressure among Myanmar migrant workers with hypertension. Furthermore, more cross-sectional studies are needed to identify barriers in promoting health literacy and to explore potential risk factors of hypertension related to behavioral and cultural adoption.

### 4.3 Strengths and limitations

To the best of our knowledge, this was the first study conducted in Northeastern Thailand, that identified the prevalence of HBP and its correlation with health literacy and other factors. Our findings revealed a protective association of health literacy to blood pressure, along with other potential risk factors. Policymakers and fellow researchers can use the outcomes of this study as a valuable reference in formulating effective public health policies and conducting relevant studies to enhance community well-being.

Regardless of our greatest efforts, there remained a few limitations to this study that should be addressed. Firstly, because the study was concentrated on registered Myanmar migrant laborers in Northeastern Thailand who had been working for at least three months duration, its results might not be entirely reflective of all migrant workers in Thailand. Secondly, to mitigate confounding bias related to health literacy in the context of healthcare, disease prevention, or health promotion activities provided by health facilities in individuals who are actively managing blood pressure with a tendency to be good health literacy, we excluded those with known hypertension who had been on antihypertensive medication for more than six months from our study. This limitation affects the generalization of our findings. Thirdly, we were unable to evaluate potential barriers to health literacy in terms of health information access, comprehension, evaluation, and implementations as we used structured questionnaire approach. Finally, due to the design of the study, we were unable to establish a causal relationship between risk factors and HBP.

## 5. Conclusions

This study discovered that more than half of Myanmar migrant workers had high blood pressure, putting them at risk of hypertension and its consequences. Limited health literacy concerning hypertension was a significant problem among Myanmar migrant workers, leading to HBP. The characteristics that were substantially linked with HBP included low health literacy by dimension of apply, being male, not having health insurance, being overnutrition and staying in Thailand for a longer period. Therefore, there is a need for a multifaceted strategy to prevent hypertension and its consequences by promoting health literacy as well as by enhancing good behavioural practice among this community.

## Supporting information

S1 File Dataset of “Health literacy and high blood pressure among Myanmar migrant workers in Northeastern Thailand”. (XLSX)
